# Contralateral prefrontal and network engagement during left DLPFC 10 Hz rTMS: an interleaved TMS-fMRI study in healthy adults

**DOI:** 10.1016/j.nicl.2025.103862

**Published:** 2025-08-06

**Authors:** Timo van Hattem, Kai-Yen Chang, Martin Tik, Paul Taylor, Jonas Björklund, Lucia Bulubas, Frank Padberg, Daniel Keeser, Mattia Campana

**Affiliations:** a Department of Psychiatry and Psychotherapy, University Hospital LMU, LMU Munich, Germany; b Neuroimaging Core Unit Munich – NICUM, University Hospital LMU, LMU Munich, Germany; c Hertie-Institute for Clinical Brain Research, University of Tübingen, Germany; d Department of Neurology & Stroke, University of Tübingen, Germany; e High Field MR Center, Center for Medical Physics and Biomedical Engineering, Medical University of Vienna, Vienna, Austria; f Brain Stimulation Lab, Department of Psychiatry and Behavioral Sciences, Stanford University, Stanford, USA; g Department of Psychology, LMU Munich, Germany; h Department of Psychology, Universität Zürich, Switzerland; i DZPG (German Center for Mental Health), partner site Munich-Augsburg, Germany; j Munich Center for Neurosciences (MCN), Ludwig Maximilian University LMU, Munich, Germany; k LVR Hospital, Department of General Psychiatry 2, Clinic of the Heinrich Heine University, Düsseldorf, Germany

**Keywords:** Interleaved TMS-fMRI, 10 Hz rTMS, iTBS, Major depression, Neuromodulation, Neuroimaging

## Abstract

**Background:**

High-frequency repetitive transcranial magnetic stimulation (rTMS) over the left dorsolateral prefrontal cortex (DLPFC) serves as an effective treatment for major depression and other psychiatric disorders. Despite its growing clinical application, the neural mechanisms by which prefrontal rTMS exerts its therapeutic effects remain incompletely understood. To address this gap, we investigated the immediate blood-oxygen-level-dependent (BOLD) activity during 600 stimuli of left DLPFC 10 Hz rTMS in healthy individuals using interleaved TMS-fMRI.

**Methods:**

In a crossover design, 17 healthy subjects received 10 Hz rTMS (60 trains with 9-second intertrain intervals) over the left DLPFC at 40 % and 80 % of their resting motor threshold (rMT) inside the MR scanner.

**Results:**

10 Hz rTMS over the left DLPFC elicited BOLD responses in prefrontal regions, cingulate cortex, insula, striatum, thalamus, as well as auditory and somatosensory areas. Notably, our findings revealed that 10 Hz rTMS effects were lateralized towards the contralateral (right) DLPFC. Dose-response effects between 40 % vs. 80 % rMT were exclusively observed in the hippocampus.

**Conclusions:**

The 10 Hz rTMS protocol used in this study induced distinct target engagement and propagation patterns in the prefrontal cortex. These patterns differ from our previous interleaved TMS-fMRI findings using 600 stimuli of left DLPFC intermittent theta burst stimulation (iTBS) at the same intensities. Thus, interleaved TMS-fMRI emerges as a valuable method for comparing clinical prefrontal rTMS protocols regarding their immediate effect on brain circuits in order to differentiate their action mechanisms and to potentially inform clinical applications.

## Highlights

• Extended 10 Hz rTMS protocols with 600 stimuli can be implemented in the interleaved TMS-fMRI setting.
• Left DLPFC 10 Hz rTMS predominantly engages contralateral DLPFC regions.
• rTMS-induced BOLD responses extend to remote salience network nodes.
• Interindividual variability in rTMS-induced BOLD responses was marked and warrants further investigation.

## 1 Introduction

Repetitive transcranial magnetic stimulation (rTMS) has emerged as an effective therapeutic intervention for various psychiatric and neurological disorders (Hyde et al., 2022, Lisanby, 2024). rTMS can modulate pathological excitability and connectivity in brain networks, resulting in symptom relief that extends beyond the immediate period of stimulation (Klomjai et al., 2015, Lefaucheur et al., 2020). High-frequency 10 Hz rTMS over the left dorsolateral prefrontal cortex (DLPFC) has become a FDA-approved clinical standard for treating pharmacoresistant major depressive disorder (MDD) (O’Reardon et al., 2007, Berlim et al., 2014, McClintock t al., 2018, Papakostas et al., 2024).

While the therapeutic benefits of rTMS are well-established (George et al., 2013, Lefaucheur et al., 2020, Kan et al., 2023), key questions persist about how rTMS engages target regions and propagates its effects through (sub)cortical networks (Siebner et al., 2022). Local and remote effects of rTMS vary depending on protocol parameters, such as stimulation frequency and intensity, with different approaches potentially recruiting distinct interneuronal circuits (Di Lazzaro et al., 2011, Hamada et al., 2013). Understanding the immediate changes in neural activity induced by rTMS across the entire brain can provide crucial insights into their underlying neurophysiological mechanisms (Tik et al., 2023a, Tik et al., 2023b, Chang et al., 2024a, Chang et al., 2024b).

Combining TMS with functional magnetic resonance imaging (fMRI) allows investigating acute effects on cerebral activation and connectivity with high spatial accuracy (Bohning et al., 1998, Bergmann et al., 2021, Mizutani-Tiebel et al., 2022). Previous TMS-fMRI research in healthy people has primarily used single-pulse TMS or short 10 Hz bursts on the primary motor cortex (M1) and DLPFC (Bergmann et al., 2021, Mizutani-Tiebel et al., 2022, Xia et al., 2024). Investigating stimulation protocols inside the MR scanner that more closely resemble rTMS protocols used in therapeutic applications could enhance the translational value of interleaved TMS-fMRI techniques by demonstrating target engagement and potentially helping identify predictive biomarkers of treatment response.

In this study, we investigate the immediate blood-oxygen-level-dependent (BOLD) responses to a 600-stimuli 10 Hz rTMS protocol at two stimulation intensities (i.e. 40 % and 80 % resting motor threshold, rMT) targeting the left DLPFC in healthy, resting individuals. This parameter selection was guided by our previous study, which used an identical interleaved TMS-fMRI setup and a similar crossover design to examine the cortical effects of a full 600-stimuli intermittent theta burst stimulation (iTBS) protocol (Chang et al., 2024a, Chang et al., 2024b). Accordingly, we also aimed to provide a descriptive comparison of the direct neural effects of 10 Hz rTMS and iTBS under matched stimulation parameters. The current study on 10 Hz rTMS, alongside the narrative comparison of its findings with iTBS, may help deepen our mechanistic understanding of how high-frequency rTMS acutely modulate local and remote neural activity, which could provide foundational knowledge to improve future neuromodulation treatments.

## 2 Methods and Materials

### 2.1 Participants

Twenty healthy subjects (13 females; mean age = 29.25 ± 7.77 years) were recruited for this study. Participants had no contraindications to TMS and MRI, and no history of psychiatric or neurological disorders. All participants provided written informed consent before the experiment. The study was approved by the ethical committee of LMU Munich and was conducted in accordance with guidelines of the Declaration of Helsinki.

Two participants dropped out during the study due to mild adverse events associated with active TMS (i.e., migraine after TMS, intolerable pressure of the TMS coil on the scalp) and one participant was excluded due to excessive motion, resulting in a total of 17 subjects (11 females; mean age = 28.18 ± 8.02 years) included for the final data analyses (Table S1).

### 2.2 Experimental design

**Fig. 1:**
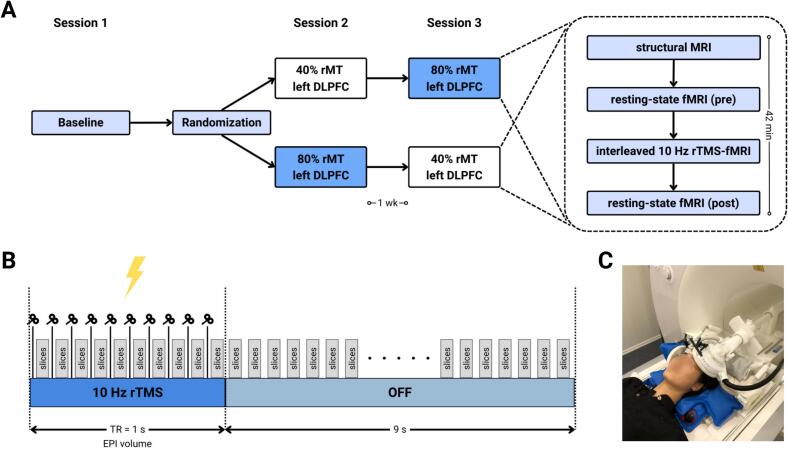
Experimental design, stimulation protocol, and setup of interleaved 10 Hz rTMS-fMRI experiment. (A) Randomized, crossover study design with a baseline measurement and two interleaved TMS-fMRI sessions. Each experimental session included structural MRI, pre- and post-resting-state fMRI, and interleaved 10 Hz rTMS-fMRI over the left DLPFC at either 40 % or 80 % of the resting motor threshold (rMT). (B) TMS pulses in 1-second trains at 10 Hz were interleaved with multi-band EPI slices, followed by 9 seconds of rest. (C) Interleaved TMS-fMRI setup with two 7-channel surface RF coils placed on each side of the head to cover the entire brain.

This study comprised a baseline measurement and two experimental sessions with interleaved TMS-fMRI (Fig. 1A). During the baseline session, subjects underwent structural MRI for neuronavigation. Individual rMT was determined for each subject while lying supine inside the MR environment. rMT was defined as the intensity that evoked a motor-evoked potential (MEP) with a peak-to-peak amplitude greater than 50 µV in five out of ten trials (Rossini et al., 1994). The average rMT was 74 % (SD = 9 %), expressed as percentage of maximal stimulator output (MSO) (Table S1). During the second and third sessions, subjects received 10 Hz rTMS over the left DLPFC inside the MR scanner with a stimulation intensity of either 40 % rMT or 80 % rMT. The intensity was randomized across sessions and the sessions were separated by a minimum of one week.

### 2.3 TMS

TMS was delivered using a MagPro X100 stimulator and a MR-compatible MRi-B91 TMS coil (MagVenture A/S, Farum, Denmark). TMS was applied over the left DLPFC (x, y, z = -38, 44, 26) (Blumberger et al., 2018) using neuronavigation (Localite GmbH, Bonn, Germany) (Fig. 1C). The 10 Hz rTMS protocol consisted of 60 trains with 10 pulses per train (i.e., interpulse interval of 0.1 s) and a 9-second intertrain interval, totaling 600 pulses per TMS-fMRI session (Fig. 1B). TMS pulses were interleaved with multi-band EPI slices. The current protocol is a modification from the standard clinical application of 10 Hz rTMS, which typically involves 3000 pulses (4 s ON, 26 s OFF) per session over 37.5 min (O’Reardon et al., 2007, Blumberger et al., 2018). Note that we reduced the number of TMS pulses to allow a direct comparison with a full iTBS protocol (i.e., 600 pulses/session) (Huang et al., 2005), such as from our previous TMS-fMRI study (Chang et al., 2024b).

### 2.4 Image acquisition

Imaging data were acquired using a 3 T Siemens PRISMA MRI-scanner (Siemens, Erlangen, Germany). T1-weighted anatomical images for neuronavigation were acquired during the baseline session using a standard 64-channel head/neck coil (TE = 2.26 ms, TR = 2300 ms, TA = 5:21 m, TI = 900 ms, flip angle = 8°, voxel size = 1.0 × 1.0 × 1.0 mm, number of slices = 192, slice thickness = 1 mm, FOV = 256 mm). During the interleaved TMS-fMRI sessions, two 7-channel surface RF coils were placed on each side of the front of the head to cover the entire brain (Fig. 1C) (Navarro de Lara et al., 2015). Structural images were acquired using a magnetization-prepared rapid gradient-echo (MP2RAGE) sequence (TE = 2.98 ms, TR = 4000 ms, TA = 6:26 m, TI = 700 ms, flip angle = 4°, voxel size = 1.0 × 1.0 × 1.0 mm, number of slices = 160, slice thickness = 1 mm, FOV = 256 mm). Multi-band accelerated echo planar imaging (EPI) sequences were used for interleaved TMS-fMRI (TE = 38  ms, TR = 1000 ms, TA = 10:27 m, flip angle = 60°, voxel size = 3.0 × 3.0 × 3.0  mm, number of slices = 40, slice thickness = 3 mm, FOV = 192 mm, MB-factor = 4).

### 2.5 Data analysis

#### 2.5.1 Preprocessing

fMRI data were preprocessed using the methods described in Chang et al., 2024a, Chang et al., 2024b). In brief, anatomical images were segmented and normalized to Montreal Neurological Institute (MNI) standard space. Functional images underwent bias-field correction, despiking, motion correction, coregistration with anatomical images, normalization, and spatial smoothing. Subjects with a mean framewise displacement greater than 0.3 mm were excluded from all further analysis (Table S1) (Power et al., 2012). An independent component analysis (ICA) was performed to reduce physiological noise (e.g., motion, cerebrospinal fluid (CSF) pulsations) and artifacts that may have been introduced by the interleaved TMS-fMRI setup (e.g, leakage currents, RF interference due to the TMS hardware) (Griffanti et al., 2017, Bergmann et al., 2021, Mizutani-Tiebel et al., 2022, Riddle et al., 2022). Data quality was checked after each preprocessing step via visual inspection. For more details, see Supplementary Materials.

#### 2.5.2 Whole-brain BOLD-fMRI

Whole-brain analysis was conducted on the denoised data in SPM12 (https://www.fil.ion.ucl.ac.uk/spm/software/spm12/) to test for brain-wide BOLD signal changes in response to 10 Hz rTMS. At the single-subject level, a linear regression was performed for each voxel using a generalized least squares method and a global approximate AR(1) autocorrelation model. A high-pass filter (128 s cut off = 0.008 Hz) using discrete cosine transform basis sets was used to remove high-frequency noise components. The regressor of interest modeled the blocked periods of stimulation at 10 Hz and was convolved with a canonical hemodynamic response function (HRF). Realignment parameters were included in the model as nuisance regressors. Individual subject-level parameter estimates (beta weights, a.u.) from the model were extracted to compute group-level averages. Statistical significance was tested against an implicit baseline with one-sample t-tests applying a p < 0.001 voxel-level threshold and a p < 0.05 Family-Wise Error (FWE) cluster-level threshold.

#### 2.5.3 Regions-of-interest

A region-of-interest (ROI) analysis was conducted to compare evoked BOLD responses across stimulation intensities in the DLPFC. A spherical ROI (radius = 10 mm) was created for the left DLPFC centered on the stimulation target (x, y, z = -38, 44, 26) (Blumberger et al., 2018, Chang et al., 2024a, Chang et al., 2024b) and for the right DLPFC on the contralateral homologous location (x, y, z = 38, 44, 26) using the MarsBaR toolbox for SPM (Brett et al., 2002). Additional spherical ROIs were generated for bilateral anterior insula, rostral ACC, and dorsal caudate nucleus as exploratory regions of interest (see Supplementary Materials). Two-way repeated measures ANOVA were performed to test for main effects of (and interaction between) intensity of stimulation and hemisphere on BOLD responses in the DLPFC. Post-hoc pairwise comparisons of mean activations in ROI were conducted using two-tailed paired t-tests (p < 0.05, FWE Holm-Bonferroni correction).

## 3 Results

### 3.1 Interleaved 10 Hz rTMS-fMRI

**Fig. 2:**
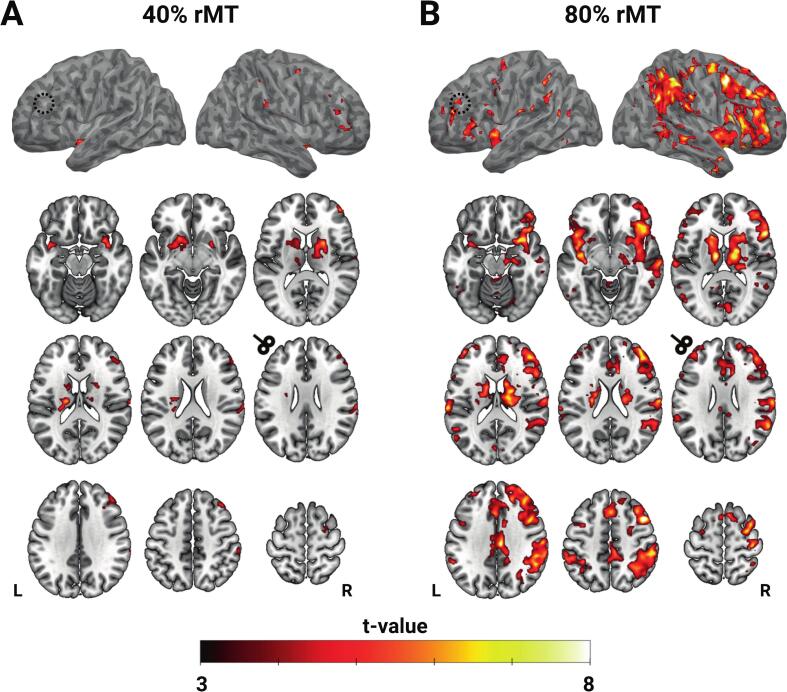
Group-level activation maps of 10 Hz rTMS-evoked BOLD responses at 40 % rMT (A) and 80 % rMT (B). All activation maps are thresholded at voxel-level p < 0.001 and cluster-level p < 0.05 FWE corrected. Axial slices display MNI Z-coordinates: −16, −8, 12; 18, 22, 26; 34, 48, 62. Dashed circle and TMS-coil pictogram roughly indicate target location.

**Fig. 3:**
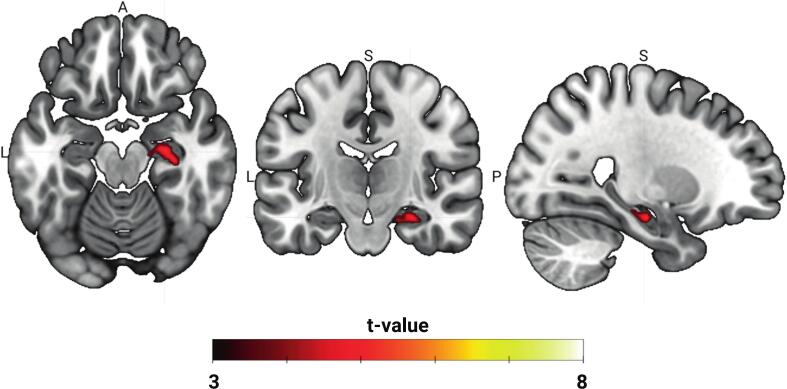
Contrast of 10 Hz rTMS-evoked BOLD responses 80 % rMT > 40 % rMT. A direct comparison of activation maps between 40 % rMT and 80 % rMT with paired *t*-test showed a cluster of significant difference in the right hippocampus (x, y, z = 26, −16, −18; k = 102; t = 5.31; Z = 3.98). Activation maps were thresholded at voxel-level p < 0.001 and cluster-level p < 0.05 FWE corrected.

A whole-brain analysis assessed BOLD activation evoked by 40 % rMT and 80 % rMT 10 Hz rTMS over the left DLPFC. Stimulation at 40 % rMT increased BOLD signal in the right middle frontal gyrus, superior frontal gyrus, and supramarginal gyrus, as well as bilateral insula, thalamus, and striatum (Fig. 2A, Table 1). No significant BOLD activation was observed at the stimulation site in the left DLPFC at 40 % rMT. At 80 % rMT, 10 Hz rTMS evoked activity in both the left and right middle frontal gyri (Fig. 2B, Table 1). Additionally, widespread BOLD activation was found in remote cortical areas, including bilateral insula, ACC, precentral gyrus, supramarginal gyrus, middle temporal gyrus, superior parietal gyrus, thalamus, and striatum. Notably, 80 % rMT stimulation evoked a stronger BOLD signal increase in the contralateral (right) hemisphere than the hemisphere stimulated. A significant difference between the two intensities on the whole-brain level was only found in the right hippocampus (Fig. 3). There was no correlation between simulated E-field strength and BOLD responses in the left DLPFC (Fig. S4).

**Table 1:** Peak BOLD activation in significant clusters during 10 Hz rTMS at 40 % rMT and 80 % rMT. Results are thresholded at voxel-level p < 0.001 and cluster-level p < 0.05 FWE corrected.

**Region**	**Hemisphere**	**Peak MNI coordinates (x, y, z)**	**t-value**	**z-value**
***40 % rMT***				
Middle frontal gyrus	R	46, 52, 12	6.73	4.57
		44, 38, 38	6.12	4.33
Superior frontal gyrus	R	24, −4, 56	6.17	4.35
Insula	L	−40, 4, −14	6.05	4.30
	R	34, 10, −16	6.07	4.31
Supramarginal gyrus	R	68, −26, 28	5.46	4.04
		58, –32, 54	5.87	4.23
Striatum	L	−20, 16, −2	6.88	4.63
	R	24, 4, 14	7.71	4.91
Thalamus	L	−18, −18, 18	6.55	4.50
	R	12, −4, 6	6.62	4.53
***80 % rMT***				
Middle frontal gyrus	L	−44, 48, 18	7.15	4.73
	R	46, 14, 48	11.42	5.88
		42, 52, 2	9.24	5.36
Superior frontal gyrus	L	−8, 10, 58	6.55	4.50
		−26, −6, 58	5.07	3.86
	R	12, 10, 70	6.70	4.56
Inferior frontal gyrus	L	−52, 28, 4	5.59	4.10
	R	56, 20, 10	10.12	5.59
Anterior/middle cingulate gyrus	L	−2, 20, 36	6.42	4.45
	R	10, 36, 18	5.30	3.97
Posterior cingulate gyrus	L	−4, −30, 42	5.85	4.22
	R	6, –22, 34	9.07	5.32
Insula	L	−42, 2, −10	6.63	4.54
	R	32, 10, −16	8.05	5.02
Precentral gyrus	R	42, −20, 60	7.32	4.78
		32, −4, 60	7.53	4.86
Supramarginal gyrus	L	−60, −42, 46	6.69	4.56
	R	58, –32, 46	8.03	5.01
Middle temporal gyrus	R	58, −28, −4	6.77	4.59
Superior temporal gyrus	L	−64, −26, 16	7.84	4.96
Superior parietal lobule	L	−38, −46, 50	6.57	4.51
	R	38, −48, 56	8.77	5.23
Striatum	L	−16, 14, 14	7.03	4.68
	R	14, 4, 20	6.63	4.53
Thalamus	L	−10, −12, 12	8.87	5.26
	R	14, −12, 14	8.85	5.26

### 3.2 DLPFC ROI analysis

**Fig. 4:**
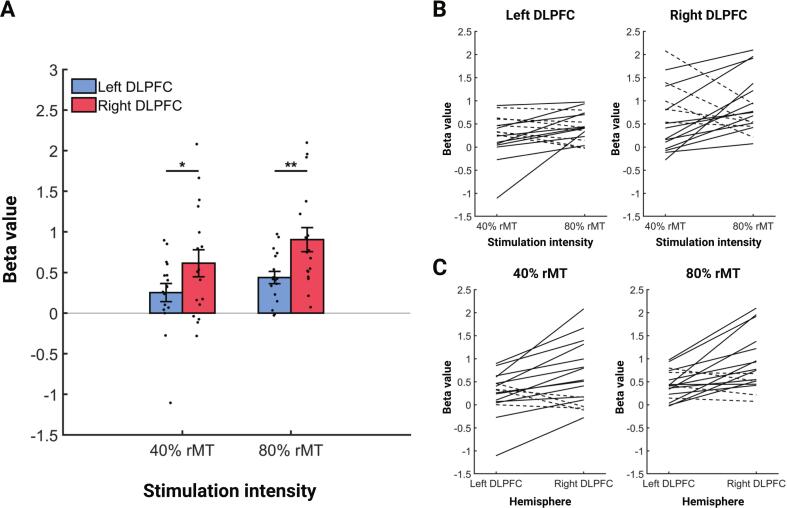
10 Hz rTMS-evoked BOLD responses in bilateral DLPFC ROIs at 40 % rMT and 80 % rMT. (A) Group-level beta value in DLPFC. Black dots represent individual subjects. Error bars show ± SEM. * p < 0.05; ** p < 0.01; *** p < 0.001. (B) Dose-response relationship in the left and right DLPFC between 40 % rMT and 80 % rMT for each subject. The solid lines indicate individuals who showed a stronger activation with 80 % rMT intensity, while the dashed lines indicate individuals who showed a stronger activation with 40 % rMT intensity. (C) Hemispheric lateralization effect in DLPFC at 40 % rMT and 80 % rMT for each subject. The solid lines indicate individuals who showed a stronger activation in the right DLPFC, while the dashed lines indicate individuals who showed a stronger activation in the left DLPFC. DLPFC = dorsolateral prefrontal cortex.

A subsequent ROI analysis was conducted to directly compare DLPFC BOLD responses evoked by 10 Hz rTMS across different stimulation intensities and to verify the observed lateralization in neural activity. We did not find a significant main effect of stimulation intensity in the DLPFC [F(1,16) = 3.194, p = 0.093]. However, there was a significant main effect of the hemisphere in the DLPFC [F(1,16) = 15.337, p = 0.001]. Post-hoc analysis revealed that BOLD signals were significantly greater in the right DLPFC than in the left DLPFC at both 40 % rMT [t(16) = -2.98, p = 0.027] and 80 % rMT [t(16) = -3.67, p = 0.008] (Fig. 4A). There were no significant interaction effects between stimulation intensity and hemispheric location of the ROI in the DLPFC. Table S3 and Table S4 summarize all descriptive and statistical values.

Responses in the bilateral DLPFC extracted from the spherical ROIs were further analysed to highlight individual responses and contributions to the group-level effects. We found that BOLD responses were increased at higher stimulation intensity in 59 % of the subjects (10/17) in the left DLPFC and in 71 % of the subjects (12/17) in the right DLPFC (Fig. 4B). Additionally, 76 % of the subjects (13/17) exhibited a greater prefrontal BOLD response in the right DLPFC compared to the left DLPFC at both 40 % rMT and 80 % rMT (Fig. 4C).

### 3.3 Temporal dynamics of whole-brain BOLD response

**Fig. 5:**
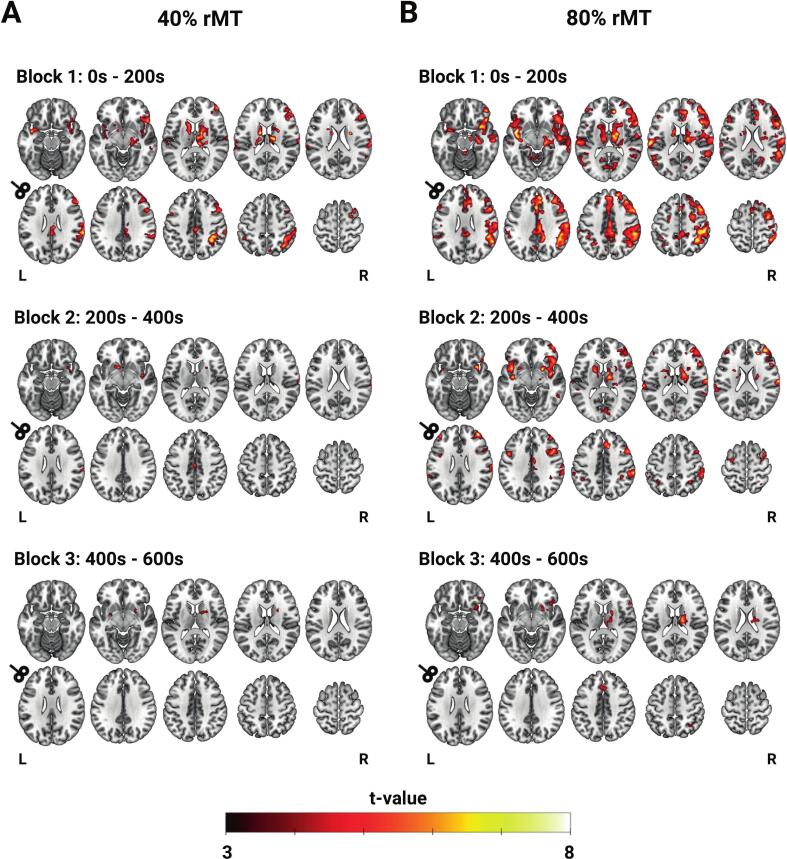
Temporal dynamics of BOLD response during 10 Hz rTMS. To examine how the BOLD response developed over time, the full 10 Hz rTMS protocol (10 min, 60 trains, 600 pulses) was divided into three blocks (each 3 min and 20 s, 20 trains, 200 pulses). (A) Whole brain activation maps across three blocks during 40 % rMT. (B) Whole brain activation maps across three blocks during 80 % rMT. All activation maps are thresholded at voxel-level p < 0.001 and cluster-level p < 0.05 FWE corrected. Axial slices have MNI Z-coordinates = –16, –8, 12, 18, 22; 26, 34, 42, 52, 62.

To examine the time-dependent cumulative effects of consecutive rTMS trains on cortical responses, we divided the 10-minute stimulation period into three equal blocks of 3 minutes and 20 seconds (200 stimuli/block), aligning with the duration of a full iTBS protocol (Chang et al., 2024b, Huang et al., 2005). In the first block, BOLD activation matched the pattern seen across the entire stimulation period, with widespread activation in the bilateral DLPFC, cingulate cortex, precentral gyrus, insula, thalamus, and striatum (Fig. 5). The second and third blocks showed a gradual decrease in whole-brain BOLD cluster activation. By the third block of 80 % rMT stimulation, significant clusters were present only in the bilateral ACC, right insula, right inferior frontal gyrus, right superior parietal lobule, right thalamus, and right striatum (both caudate nucleus and putamen) (Fig. 5B). The second and third block of 40 % rMT were characterized by significant bilateral striatal activation (both putamen and right caudate) (Fig. 5A). Similar effects were seen when dividing blocks into six blocks of 100 pulses (Fig. S5). To rule out the possibility of visualization artifacts from the implicit baseline, the group-average ROI beta values across time windows are also presented in the Supplementary Materials. Overall, BOLD activity initially plateaued, followed by a modest reduction over time (Fig. S5).

## 4 Discussion

The present study aimed to characterize neural responses to a 10 Hz rTMS protocol over the left DLPFC in healthy individuals using interleaved TMS-fMRI. We found that 10 Hz rTMS elicited BOLD responses in prefrontal regions, ACC, insula, striatum, thalamus, as well as auditory and somatosensory areas. Notably, the 10 Hz rTMS effects in the DLPFC were lateralized towards the contralateral (right) DLPFC. Dosage-dependent responses were only found in the right hippocampus. Compared to our previous interleaved TMS-fMRI study applying iTBS over the left DLPFC with identical pulse numbers and intensities (Chang et al., 2024b), the 10 Hz rTMS effects showed distinct patterns of activation.

### 4.1 rTMS-evoked brain activity patterns

Previous interleaved 10 Hz TMS-fMRI studies of prefrontal regions in healthy subjects typically used shorter 10 Hz bursts (i.e., 3–8 pulses) and fewer total pulses per experimental condition (Hawco et al., 2017, Caparelli et al., 2022, Tik et al., 2023a, 2023b). The 10 Hz rTMS protocol in the current study with 600 stimuli (1 s ON, 9 s OFF) represents the longest application of a prefrontal 10 Hz rTMS protocol inside a MR scanner to date and parallels a full 600-stimuli iTBS protocol (Huang et al., 2005, Chang et al., 2024b).

We found that 10 Hz rTMS modulated neural activity within the target DLPFC and in interconnected cortical and subcortical regions. The observed BOLD signal increases in areas such as the ACC, insula, striatum, and thalamus suggest modulation of the salience network (SN). Earlier TMS-fMRI work stimulating the left DLPFC during rest with either single pulses or 10 Hz bursts reported similar patterns in distal network-wide activation (Hanlon et al., 2013, Vink et al., 2018, Dowdle et al., 2018, Tik et al., 2023a, 2023b). While both the SN and the Default Mode Network (DMN) are frequently implicated in the broader network effects of TMS in both clinical and healthy populations, our findings indicate consistent immediate engagement of SN nodes during 10 Hz rTMS. DMN involvement was less pronounced, suggesting that the SN may be a more direct and sensitive target of target engagement under this stimulation paradigm. This aligns with evidence highlighting the SN’s key role in MDD, where its dysfunction has been linked to symptomatology and treatment response (Downar et al., 2016, Seeley, 2019). It should be noted, however, that activation of SN regions may partly reflect auditory or somatosensory responses to the TMS pulse, as well as anticipation and attentional effects (Iannetti and Mouraux, 2010, Dowdle et al., 2018). Future studies should further disentangle these contributions and clarify the distinct roles of these networks, particularly concerning clinical outcomes.

In our study, while we did observe a small cluster of activation at the stimulation site (Fig. 2), the dominant and more reproducible finding was contralateral DLPFC activation. The absence of strong BOLD activation directly beneath the TMS coil remains an ongoing topic of debate, with mixed findings reported in the literature (Bergmann et al., 2021, Rafiei and Rahnev, 2022). Several studies have observed robust BOLD activity under the coil when stimulating M1 or V1, but these effects may be driven by the downstream consequences of TMS (e.g., muscle twitches) (Rafiei & Rahnev, 2022). For other areas, such as DLPFC, TMS does not appear to increase BOLD activity at the site of stimulation when conducted at rest (Rafiei and Rahnev, 2022, Xia et al., 2024). Other studies using comparable protocols have reported distributed or contralateral effects rather than focal activation at the stimulation site, suggesting that DLPFC stimulation engages large-scale prefrontal networks and subcortical circuits rather than eliciting strong local BOLD responses (e.g., Bergmann et al., 2021, Oathes et al., 2021). Our findings align with previous studies using similar interleaved TMS-fMRI designs targeting the left DLPFC by Tik et al. (2023a, 2023b), who demonstrated that brief 10 Hz TMS triplets applied at higher intensities (80–110 % rMT) consistently engaged the right DLPFC more strongly than the area directly beneath the coil. This network-level engagement across varying intensities aligns with our own observation of contralateral prefrontal activation. Prior work (e.g., Beynel et al., 2020) has shown that the network-level impact of TMS is strongly shaped by the frequency, intensity, and duration of stimulation, as well as the cognitive state during stimulation. We hypothesize that 10 Hz rTMS at lower intensities preferentially activates transcallosal or network-level pathways, while higher intensities (or longer stimulation duration) may be needed to elicit stronger local effects. This may differ from other protocols, such as iTBS, where the high pulse frequency might drive local activation even at lower intensities (Chang et al., 2024a, Chang et al., 2024b).

It was evident that group-level effects were strongly driven by the first few minutes of the 10-minute stimulation protocol. Notably, local activity was observed only during the first block, while activation shifted to remote areas as the protocol progressed (Fig. 5; Fig. S5). Our findings point to robust fronto-striatal-thalamic network engagement during left DLPFC 10 Hz rTMS, as these were stable across all temporal windows for 80 % rMT as well as 40 % rMT. Previous research has identified strong structural and functional connections between the DLPFC and striatal areas (Leh et al., 2007, Di Martino et al., 2008). Moreover, rTMS over the DLPFC modulates subcortical dopaminergic neurotransmission in healthy subjects (Strafella et al., 2001) and in those with depression (Pogarell et al., 2006, Pogarell et al., 2007). Accordingly, the frontal-striatal-thalamic pathway may play a key role in the antidepressant effect of rTMS in depression (Avissar et al., 2017). In future studies, interleaved TMS-fMRI in clinical populations may be therefore used to elucidate whether fronto-striatal-thalamic network engagement predicts better clinical response to rTMS. Another open question is to what extent such initial local and sustained remote effects during 10 Hz rTMS contribute to its therapeutic efficacy. A deeper understanding of both temporal dynamics and interindividual variance of effects may guide choices of stimulation patterns and durations required to achieve optimal neurophysiological effects and clinical outcomes.

Due to the large interindividual variability in TMS effects, stronger responders may disproportionately contribute to the observed hemispheric lateralization. However, our analyses show that DLPFC effects remain robust after correcting for potential outliers (see Supplementary Materials), though their influence cannot be entirely excluded. Replication in larger, independent samples will be crucial to confirm and extend these findings. Future studies should further examine whether observed variability in local and downstream engagement is mediated by functional or anatomical connectivity at the stimulation site (e.g., in the prefrontal-hippocampal network). Here, stimulation intensity was based on motor threshold without depth correction for the DLPFC, so anatomical differences – such as skull-to-cortex distance – may have contributed to BOLD variability and the lack of correlation with simulated E-field strength. Individualized, anatomy-based dosing in future work could help reduce this variability and improve targeting precision.

### 4.2 High and low intensity of 10 Hz rTMS

The field of TMS lacks an established sham control capable of fully isolating the neurophysiological effects of TMS independently of accompanying somatosensory responses (Arana et al., 2008, Duecker and Sack, 2015, Bagali et al., 2023). In this study, we employed an active intensity control at 40 % rMT, similar to our previous study (Chang et al., 2024b). This study comprised separate sessions for each intensity, whereas previous TMS-fMRI studies often compared multiple stimulation intensities within a single session (Nahas et al., 2001, Baudewig et al., 2001, Moisa et al., 2010, Tik et al., 2023a, Tik et al., 2023b), which may have led to carry-over effects and reduced signal-to-noise ratio (Seewoo et al., 2019).

In the left DLPFC target region, we did not observe a dose-dependent BOLD effect comparing 40 % and 80 % rMT intensity. While linear TMS dose-responses are consistently detected in M1 (Bohning et al., 1999, Hanakawa et al., 2009, Navarro de Lara et al., 2017, Chang et al., 2024b), findings from studies using interleaved TMS-fMRI over the left DLPFC are mixed regarding dose-dependent effects (Nahas et al., 2001, Dowdle et al., 2018, Tik et al., 2023b, Chang et al., 2024b). Only 59 % of the subjects in our study showed increased left DLPFC activity with higher stimulation intensity, potentially preventing a group-level effect. Similarly, previous inconclusive findings may have been due to the large between-subject variability (Tik et al., 2023b). The variability across participants likely reflects a combination of neurophysiological and methodological factors. First, variations in cortical folding and depth of the DLPFC due to scalp-to-cortex distance across individuals can affect the effective electric induced field delivered to the brain, leading to inconsistent stimulation even when the same percentage of rMT is used. Small differences in targeting due to differences in coil positioning and angle, may have influenced the effective induced current across sessions, resulting in different physiological effects across subjects and within-subjects across sessions. Second, the relationship between stimulation intensity and BOLD response may not necessarily be linear in the DLPFC (Tik et al., 2023b). At different stimulation intensities, different subpopulations of neurons may be recruited. Some individuals may reach a plateau or even exhibit suppression at higher intensities due to homeostatic or inhibitory mechanisms (e.g., activation of inhibitory interneurons at higher thresholds). Lastly, pre-stimulus brain states (e.g., fluctuations in arousal, attention, or ongoing oscillatory activity) can shape TMS-evoked responses (e.g., higher prefrontal alpha power is linked to reduced evoked responses to TMS) (Bradley et al., 2022). Such dynamic states may affect individual sensitivity to increased stimulation intensity.

Dose-response effects (40 % vs. 80 % rTMS) at the whole-brain level were exclusively observed in the hippocampus. The DLPFC and hippocampus are known to be functionally and anatomically connected via both direct and indirect pathways (Goldman-Rakic et al., 1984). Alpha (8–12 Hz) and theta (4–7 Hz) oscillations may coordinate memory processes of the prefrontal-hippocampal network (Herweg et al., 2016, Backus et al., 2016). The hippocampus may be susceptible to changes in cortical excitability, exhibiting network-level effects of prefrontal 10 Hz rTMS due to its susceptibility to oscillatory coupling, especially in the theta and alpha bands (Kaiser et al., 2025). Furthermore, prior research has reported that remote areas rather than the stimulated DLPFC show compensatory patterns to rTMS, with reported linear activity increases in subcortical regions with increasing stimulation intensities of rTMS over left DLPFC (Tik et al., 2023b), potentially reflecting more effective downstream propagation of rTMS effects to subcortical regions. The absence of further significant differences at the cortical level between the two intensities despite broader engagement at 80 % RMT, may be the result of large interindividual variability, differences in baseline connectivity, or statistical power limitations in whole-brain comparisons. Future work should explore whether connectivity-guided targeting can enhance downstream engagement and individualize stimulation effects, potentially improving consistency and efficacy of network-level modulation.

### 4.3 Comparing outcomes of 10 Hz rTMS and iTBS with interleaved TMS-fMRI

There was significant lateralization of effects during left DLPFC 10 Hz rTMS towards the contralateral homologous region. This was evident in most subjects (Fig. 4), and persisted throughout the entire stimulation protocol (Fig. 5, Fig. S5). Previous TMS-fMRI (Tik et al., 2023b) and TMS-EEG studies (Ye et al., 2022, Ross et al., 2023) have similarly shown evidence that left prefrontal 10 Hz rTMS has its strongest effects in the right hemisphere. Activation of distant regions could theoretically be due to nonspecific effects associated with TMS-fMRI, and it may be tempting to expect and interpret effects in proximal regions as more stimulation target related than effects in distal regions. Second, cortical stimulation specific effects could be explained by transsynaptic effects of target modulation, but it is not clear whether these distant effects are due to real excitatory stimulation (as often assumed) or mediated by interference with the complex inhibitory control of cortical microcircuits (Hamada et al., 2013).

Interestingly, the present findings largely differ from our previous findings of left DLPFC stimulation by a 600-stimuli iTBS protocol at 40 % and 80 % rMT, where the BOLD activation in the left DLPFC was significantly higher than in the right DLPFC (Chang et al., 2024b). Regarding their clinical applications, iTBS, which is a patterned high-frequency rTMS protocol delivering bursts of 50 Hz triplets repeated at 5 Hz (Huang et al., 2005), and 10 Hz rTMS, are often regarded as equivalent in terms of their therapeutic effects in MDD (Blumberger et al., 2018, Bulteau et al., 2022, Spitz et al., 2022). In direct comparison, however, the neurophysiological action of these two widely used stimulation protocols for MDD – 10 Hz rTMS and iTBS – remain largely unexplored, and their clinical equivalence has been recently challenged (Li et al., 2023, Wada et al., 2025). The findings of both interleaved TMS-fMRI studies together suggest that 10 Hz rTMS and iTBS are protocols with evidently distinct neurophysiological properties in terms of target engagement and propagation patterns. These protocols differed only in how the TMS pulses are grouped together over time, which means that the timing of TMS pulses could play a pivotal role in determining acute brain responses, possibly by recruiting distinct neuronal populations.

Early findings for M1 (Pascual-Leone et al., 1994, Wassermann et al., 1998, Huang et al., 2005) have led to the misguided assumption that rTMS protocols are inherently physiologically excitatory (when the stimulation frequency is higher than 5 Hz) or inhibitory (when the frequency is lower than 5 Hz) in their cortical effects, even when they are applied to non-motor cortical sites such as the DLPFC (Hussain & Freedberg, 2025). Indeed, neuromodulatory effects of standard rTMS in motor regions may be less robust than initially expected, despite the fact that these protocols were originally established for motor system plasticity induction. Recent studies investigating common variants of rTMS over M1 showed highly variable interindividual and intraindividual effects with little consistency (Schilberg et al., 2017, Boucher et al., 2021, Magnuson et al., 2023).

While left DLPFC activation progressively increased over the 3-minute iTBS protocol (Chang et al., 2024a), we found no such cumulative effect with 10 Hz rTMS. Strikingly, iTBS and 10 Hz rTMS produced similar activation patterns at the lower intensity: the higher frequency (or shorter duration) of iTBS may induce plasticity underneath the coil more effectively. Whether 10 Hz rTMS would require higher intensities or longer durations (such as in its clinical use) (O’Reardon et al., 2007) to achieve similar cumulative effects remains speculative and warrants further research.

### 4.4 Limitations & future directions

Simultaneous TMS and fMRI acquisition is technically challenging and inherently introduces rigid artifacts into the imaging data (Mizutani-Tiebel et al., 2022). In addition, prolonged rTMS inside the MR scanner (i.e., ∼10 min for 10 Hz rTMS) can cause substantial discomfort compared to offline rTMS, leading to increased head motion and lower signal-to-noise. While a larger sample size would potentially improve the robustness and reliability of the findings, the high costs associated with TMS-fMRI data acquisition need to be considered.

In this study, we modified the standard therapeutic 10 Hz rTMS protocol (100 %-120 % rMT; 4 s ON, 26 s OFF; 3000 pulses; 37.5 min) (O’Reardon et al., 2007), implementing subthreshold intensities (40 % and 80 % rMT) and shorter duration (600 pulses, 10 min), for three primary reasons: 1) technical constraints of the TMS coil (e.g., heating, larger MR artifacts), 2) reducing participant discomfort, and 3) facilitating a direct comparison with the full iTBS protocol (i.e., 600 pulses/session and 80 % MT), as it was also implemented in prior work by Chang et al. (2024b). Further advancements in interleaved TMS-fMRI technology ought to enable closer alignment with both offline and online therapeutic rTMS protocols in the future. Although 80 % rMT is below standard clinical dosing (∼110 % rMT), our intensity range was selected to explore the physiological response space. These findings provide mechanistic insights that warrant further investigation at clinically relevant intensities in future translational studies. Whereas the inclusion of more suprathreshold stimulation intensities (Tik et al., 2023b) or other stimulation targets, e.g., M1 (Chang et al., 2024b), would have allowed us to investigate the specificity of effects observed here, this was generally restricted by these experimental limitations. Including a spatial control condition (e.g., vertex or M1) would have substantially strengthened the interpretability of our findings by helping to dissociate site-specific neural engagement from general intensity-related effects. However, incorporating such a control was beyond the scope of the current study due to resource and scan time constraints. We think it is critical that future studies consider both spatial control conditions, ideally alongside discomfort ratings, to more rigorously isolate network-specific effects of prefrontal rTMS.

Importantly, these findings in a healthy population may have limited translation value and applicability to clinical populations. rTMS may affect brain activity differently in individuals with pathological brain functions. Extending TMS-fMRI research to clinical populations with network disorders (e.g., MDD, schizophrenia, obsessive–compulsive disorder) is necessary to establish clinical relevance and determine its potential in identifying predictive biomarkers for neuromodulation-based treatment in psychiatry.

Given the absence of a sham control or control site, we cannot fully disentangle neural effects of 10 Hz rTMS from non-specific factors such as auditory and somatosensory stimulation, discomfort, or expectancy, which may partially drive salience network activation (e.g., in the ACC, insula, or putamen). The absence of a control site or sham condition currently limits the interpretability of these effects.

## 5 Conclusions

Using interleaved TMS-fMRI, this study provides evidence of distinct prefrontal target engagement and propagation patterns during left prefrontal 10 Hz rTMS. By comparing the current findings on 10 Hz rTMS to previous findings from our lab on iTBS, this leads us to speculate that distinct neurophysiological mechanisms may be involved in 10 Hz rTMS and iTBS that determine the propagation of stimulation effects to interconnected brain regions. While our findings suggest differing patterns of activation between 10 Hz rTMS and previously reported iTBS effects, these interpretations remain preliminary given the between-study design and known individual variability in TMS responses. Further research with within-subject comparisons is necessary to elucidate the underlying mechanisms of these therapeutic rTMS protocols and to determine how acute effects captured with TMS-fMRI relate to treatment response in clinical populations. Future experiments with proper spatial sham controls are also needed to validate the neural effects and disentangle them from potential auditory and somatosensory confounds. In sum, interleaved TMS-fMRI using full rTMS protocols represents a highly promising approach for investigating rTMS protocols in terms of their immediate effects on brain circuits including individual differences which could lead to the development of clinically effective and personalized rTMS in the future.

## Funding information

This project was funded by the Federal Ministry of Education and Research within the ERA-NET NEURON program (Grant No. FKZ: 01EW1903: DisCoVeR) and the German Center for Mental Health (Grant No. 01EE2303, project MUC6). The procurement of the Prisma 3T MRI scanner was supported by the Deutsche Forschungsgemeinschaft (Grant No. INST 86/1739-1 FUGG). LB’s work was part of the funding program of the Medical Faculty of LMU (FöFöLe Plus, Munich Clinician Scientist Program). MT’s travel costs and research stays were supported by the visiting scholarship artificial intelligence programme (BaCaTeC 2022/01, Machine learning-based optimization for personalized brain stimulation therapy). PT’s work was funded by the Deutsche Forschungsgemeinschaft (Grant No. TA 857/3-2). MC’s work was part of the funding program of the Medical Faculty of LMU (FöFöLe, Munich Clinician Scientist Program). TvH is funded by the European Research Council (ERC Synergy) under the European Union's Horizon 2020 research and innovation program (ConnectToBrain, grant number 810377).

## CRediT authorship contribution statement

**Timo van Hattem:** Writing – review & editing, Writing – original draft, Visualization, Project administration, Methodology, Investigation, Formal analysis, Data curation, Conceptualization. **Kai-Yen Chang:** Writing – review & editing, Validation, Supervision, Project administration, Methodology, Investigation, Conceptualization. **Martin Tik:** Writing – review & editing, Validation, Methodology, Conceptualization. **Paul Taylor:** Writing – review & editing. **Jonas Björklund:** Writing – review & editing. **Lucia Bulubas:** Writing – review & editing, Project administration. **Frank Padberg:** Writing – review & editing, Validation, Supervision, Resources, Project administration, Methodology, Funding acquisition, Conceptualization. **Daniel Keeser:** Writing – review & editing, Validation, Supervision, Resources, Project administration, Methodology, Funding acquisition, Conceptualization. **Mattia Campana:** Writing – review & editing, Validation, Supervision, Project administration, Methodology, Investigation, Funding acquisition, Conceptualization.

## Declaration of competing interest

The authors declare that they have no known competing financial interests or personal relationships that could have appeared to influence the work reported in this paper.

## Data Availability

Access to the completed MRI data and unthresholded statistical maps are available upon request. The MRI can be obtained upon formalizing a data sharing agreement, though not publicly accessible due to challenges in achieving complete anonymization.
