# Supplementary material for: Contralateral prefrontal and network engagement during left DLPFC 10 Hz rTMS: an interleaved TMS-fMRI study in healthy adults
Source: Neuroimage Clin. 2025 Aug 6;48:103862. doi: 10.1016/j.nicl.2025.103862 (PMC12362399; doi:10.1016/j.nicl.2025.103862)
Supplement: Supplementary Data 1 [file mmc1.docx]

**Appendix. Supplementary Methods**

**fMRI preprocessing**

Data preprocessing and statistical analysis were performed using MATLAB (Mathworks, Natrick, MA) and the SPM12 toolbox (Wellcome Trust Centre for Neuroimaging, London, United Kingdom), as well as ANFI (http://afni.nimh.nih.gov/afni) and ANTs (http://stnava.github.io/ANTs). Preprocessing of the fMRI data included the following steps: 1) segmentation and normalization of the anatomical images to Montreal Neurological Institute (MNI) standard space with deformation fields using CAT12 (http://www.neuro.uni-jena.de/cat/); 2) bias-field correction of the functional images with N4BiasFieldCorrection implemented in ANTs; 3) despiking of the functional images using ANFI; 4) realignment of the functional images (SPM12); 5) coregistration of the functional images to the anatomical images (SPM12); 6) normalization of coregistered functional images to MNI standard space based on deformation fields derived from anatomical normalization (SPM12); and 7) spatial smoothing using a 6 mm FNWH Gaussian kernel (SPM12). Subjects with mean framewise displacement greater than 0.3 mm were excluded from all further analysis (Power et al., 2012). Data quality was checked after each preprocessing step via visual inspection.

In order to reduce physiological noise (e.g., motion, cerebrospinal fluid (CSF) pulsations) and artifacts that may have been introduced by the interleaved TMS-fMRI setup (e.g, leakage currents, mechanical vibrations, RF interference due to the TMS hardware) (Bergmann et al., 2021; Riddle et al., 2022; Mizutani-Tiebel et al., 2022), an independent component analysis (ICA) was performed using FSL MELODIC (Jenkinson et al., 2012). Time signals were decomposed into 25 components and denoised via manual rejection (Griffanti et al., 2017). An average of 20.42 (SD = 1.52) components were removed across both conditions.

**Exploratory network-level ROI analysis: Insula, ACC, Caudate**

Aside from the DLPFC ROIs, spherical ROIs were also generated for bilateral anterior insula, rostral ACC, and dorsal caudate nucleus as exploratory regions of interest. The analysis further explored downstream network-level engagement from left DLPFC stimulation. These ROIs were selected *a priori* based on previous work indicating that left DLPFC stimulation activates interconnected salience and striatal networks (Hanlon et al., 2013; Hawco et al., 2018; Riddle et al., 2022).

Their sizes were modified where necessary to prevent anatomical overlap. We initially defined spherical ROIs with a 10 mm radius, consistent with the size of the DLPFC ROI and with our previous work (Chang et al., 2024a, 2024b). However, for the rostral ACC and dorsal caudate, this size resulted in overlap with adjacent structures. To maintain anatomical plausibility, the sphere radius for these regions was reduced to 5 mm. See Table S2 and Figure S1 for sphere radius and MNI coordinates of center of mass for each ROI. Given the exploratory nature of these additional ROIs, no correction for multiple comparisons based on the total number of ROIs was applied.

We observed a significant main effect of stimulation intensity bilaterally in the insula [F(1,16) = 10.182, p = 0.006], but not in the ACC [F(1,16) = 3.653, p = 0.074], nor in the caudate [F(1,16) = 0.735, p = 0.404]. Specifically, BOLD activation was significantly greater at 80% rMT than 40% rMT in both the left insula [t(16) = -3.01, p = 0.032] and right insula [t(16) = -2.87, p = 0.033] (Figure S2). Additionally, there was a significant main effect of the hemisphere in the insula [F(1,16) = 6.518, p = 0.021]. Post-hoc analysis revealed that BOLD signals were significantly greater in the right insula than in the left insula at 80% rMT [t(16) = -2.50, p = 0.048] (Figure 2). There were no significant interaction effects between stimulation intensity and hemispheric location of ROIs. Table S3 and Table S4 summarize all descriptive and statistical values.

**E-field modeling**

The open-source software package SimNIBS v4.1.0 was used for realistic calculations of the electric field (E-field) induced by TMS (Thielscher et al., 2015). High-resolution anatomical T1w and T2w images (TE = 383 ms, TR = 5000 ms, TA = 4:57 m, TI =1800 ms, flip angle = 8°, voxel size = 1.0 × 1.0 × 1.0 mm, number of slices = 176, slice thickness = 1 mm, FOV = 256 mm) from the baseline session were segmented into gray matter, white matter, and CSF, to construct realistic head models for each subject. Using stimulation intensity output values from the TMS stimulator (di/dt = A/µs), the individualized E-field on the cortex was simulated for each session. The location and orientation of the TMS coil were derived from the first markers recorded by the neuronavigation system. Three subjects were excluded in the 40% rMT (n = 14) condition and two subjects in the 80% rMT (n = 15) condition for E-field analysis because stimulation markers or intensity values were not properly recorded. Mean E-field strength in the left DLPFC was extracted using a spherical ROI (radius = 10 mm, x, y, z = -38, 44, 26) and correlated to mean ROI beta values in left DLPFC (Pearson’s correlation, p < .05).

The E-field induced by 10 Hz rTMS was focally located at the target site in the left DLPFC at 40% rMT (M = 28.12 V/m, SD = 6.85) and 80% rMT (M = 57.84 V/m, SD = 10.10) [paired t-test: t(12) = 14.662, p < .001] (Figure S3). However, there was a lack of statistically significant correlations between E-field strength and individual BOLD signal in the left DLPFC at both 40% rMT [r = -0.077, p = 0.793] and 80% rMT [r = 0.363, p = 0.184] (Figure S4).

**Appendix. Supplemental Tables**

**Table S1. Subject demographics and mean framewise displacement.**

**Table S2. Location and spherical size of regions-of-interest (ROI).**

**Table S3. 10 Hz rTMS descriptive statistics for each ROI.**

**Table S4. 10 Hz rTMS test statistics for each ROI.**

**Table S1.** Subject demographics and mean framewise displacement. Framewise displacement was calculated according to Power et al. (1)

| **Subject ID** | **Age** | **Sex** | **rMT (% MSO)** | **Mean FD - 40% rMT (mm)** | **Mean FD - 80% rMT (mm)** |
| --- | --- | --- | --- | --- | --- |
| sub001 | F | 24 | 78 | 0.10 | 0.18 |
| sub002 | M | 56 | 69 | 0.21 | 0.12 |
| sub003 | F | 24 | 74 | 0.10 | 0.11 |
| sub005 | M | 32 | 66 | 0.06 | 0.07 |
| sub007 | F | 31 | 72 | 0.10 | 0.18 |
| sub008 | F | 28 | 80 | 0.09 | 0.09 |
| sub009 | F | 21 | 71 | 0.10 | 0.12 |
| sub010 | F | 25 | 70 | 0.09 | 0.11 |
| sub011 | M | 33 | 75 | 0.09 | 0.10 |
| sub012 | F | 26 | 89 | 0.11 | 0.09 |
| sub014 | M | 31 | 71 | 0.09 | 0.12 |
| sub015 | F | 27 | 61 | 0.09 | 0.10 |
| sub016 | M | 23 | 70 | 0.17 | 0.15 |
| sub017 | F | 24 | 75 | 0.09 | 0.12 |
| sub018 | F | 28 | 68 | 0.09 | 0.12 |
| sub019 | F | 23 | 87 | 0.10 | 0.13 |
| sub020 | M | 23 | 90 | 0.15 | 0.12 |

**Table S2.** Location and spherical size of regions-of-interest (ROI).

| **ROI** | **Left hemisphere MNI coordinates (x, y, z)** | **Right hemisphere MNI coordinates (x, y, z)** | **Radius sphere (mm)** | **Reference** |
| --- | --- | --- | --- | --- |
| DLPFC | -38, 44, 26 | 38, 44, 26 | 10 | (Blumberger et al., 2018) |
| Rostral ACC | -5, 34, 28 | 5, 34, 28 | 5 | (Zhou et al., 2016) |
| Anterior insula | -44, 13, 1 | 47, 14, 0 | 10 | (Krönke et al., 2020) |
| Dorsal caudate nucleus | -13, 15, 9 | 13, 15, 9 | 5 | (Park et al., 2020; Peng et al., 2022) |

**Table S3**. 10 Hz rTMS descriptive statistics for each ROI.

| **ROI** | **Hemisphere** | **Mean** | **Standard deviation** |
| --- | --- | --- | --- |
| ***40% rMT*** | | | |
| DLPFC | L | 0.253 | 0.464 |
|  | R | 0.614 | 0.686 |
| ACC | L | 0.518 | 0.996 |
|  | R | 0.677 | 1.140 |
| Insula | L | 0.362 | 0.969 |
|  | R | 0.609 | 1.440 |
| Caudate | L | 0.840 | 0.937 |
|  | R | 0.793 | 1.020 |
| ***80% rMT*** | | | |
| DLPFC | L | 0.438 | 0.312 |
|  | R | 0.905 | 0.614 |
| ACC | L | 0.928 | 0.788 |
|  | R | 1.100 | 1.070 |
| Insula | L | 1.110 | 0.776 |
|  | R | 1.700 | 1.510 |
| Caudate | L | 0.975 | 0.820 |
|  | R | 1.050 | 0.856 |

**Table S4**. 10 Hz rTMS test statistics for each ROI.

|  | **Test statistic** | **p-uncorrected** | **p-corrected** | **Significance** |
| --- | --- | --- | --- | --- |
| ***DLPFC*** | | | | |
| Main effect: intensity | F(1,16) = 3.194 | 0.093 | - | - |
| Main effect: hemisphere | F(1,16) = 15.337 | 0.001 | - | ***** |
| Interaction effect | F(1,16) = 0.659 | 0.429 | - | - |
| left 40% rMT - left 80% rMT | t(16) = -1.76 | 0.098 | 0.196 | - |
| right 40% rMT - right 80% rMT | t(16) = -1.60 | 0.128 | 0.196 | - |
| left 40% rMT - right 40% rMT | t(16) = -2.98 | 0.009 | 0.027 | ***** |
| left 80% rMT - right 80% rMT | t(16) = -3.67 | 0.002 | 0.008 | ****** |
| ***ACC*** | | | | |
| Main effect: intensity | F(1,16) = 3.653 | 0.074 | - | - |
| Main effect: hemisphere | F(1,16) = 1.340 | 0.264 | - | - |
| Interaction effect | F(1,16) = 0.007 | 0.932 | - | - |
| left 40% rMT - left 80% rMT | t(16) = -1.92 | 0.073 | 0.292 | - |
| right 40% rMT - right 80% rMT | t(16) = -1.65 | 0.118 | 0.354 | - |
| left 40% rMT - right 40% rMT | t(16) = -1.03 | 0.320 | 0.640 | - |
| left 80% rMT - right 80% rMT | t(16) = -0.947 | 0.358 | 0.640 | - |
| ***Insula*** | | | | |
| Main effect: intensity | F(1,16) = 10.182 | 0.006 | - | ***** |
| Main effect: hemisphere | F(1,16) = 6.518 | 0.021 | - | ***** |
| Interaction effect | F(1,16) = 1.466 | 0.244 | - | - |
| left 40% rMT - left 80% rMT | t(16) = -3.01 | 0.008 | 0.032 | ***** |
| right 40% rMT - right 80% rMT | t(16) = -2.87 | 0.011 | 0.033 | ***** |
| left 40% rMT - right 40% rMT | t(16) = -1.26 | 0.226 | 0.226 | - |
| left 80% rMT - right 80% rMT | t(16) = -2.50 | 0.024 | 0.048 | ***** |
| ***Caudate*** | | | | |
| Main effect: intensity | F(1,16) = 0.735 | 0.404 | - | - |
| Main effect: hemisphere | F(1,16) = 0.018 | 0.895 | - | - |
| Interaction effect | F(1,16) = 0.753 | 0.398 | - | - |
| left 40% rMT - left 80% rMT | t(16) = -0.636 | 0.534 | 1 | - |
| right 40% rMT - right 80% rMT | t(16) = -0.979 | 0.342 | 1 | - |
| left 40% rMT - right 40% rMT | t(16) = 0.390 | 0.702 | 1 | - |
| left 80% rMT - right 80% rMT | t(16) = -0.642 | 0.530 | 1 | - |

*Note.* P-values are FWE corrected. * p < .05; ** p < .01; *** p < .001

**Appendix. Supplemental Figures**

**Figure S1. Location and spherical size of regions-of-interest (ROI).**

**Figure S2. 10 Hz rTMS-evoked BOLD responses in bilateral Insula, ACC, Caudate ROIs at 40% rMT and 80% rMT.**

**Figure S3. 10 Hz rTMS-evoked BOLD responses in bilateral ROIs at 40% rMT and 80% rMT with outlier removal.**

**Figure S4. E-field simulations of 10 Hz rTMS over the left DLPFC.**

**Figure S5. Temporal dynamics of BOLD response during 10 Hz rTMS protocol.**

**Figure S6. Distress ratings.**


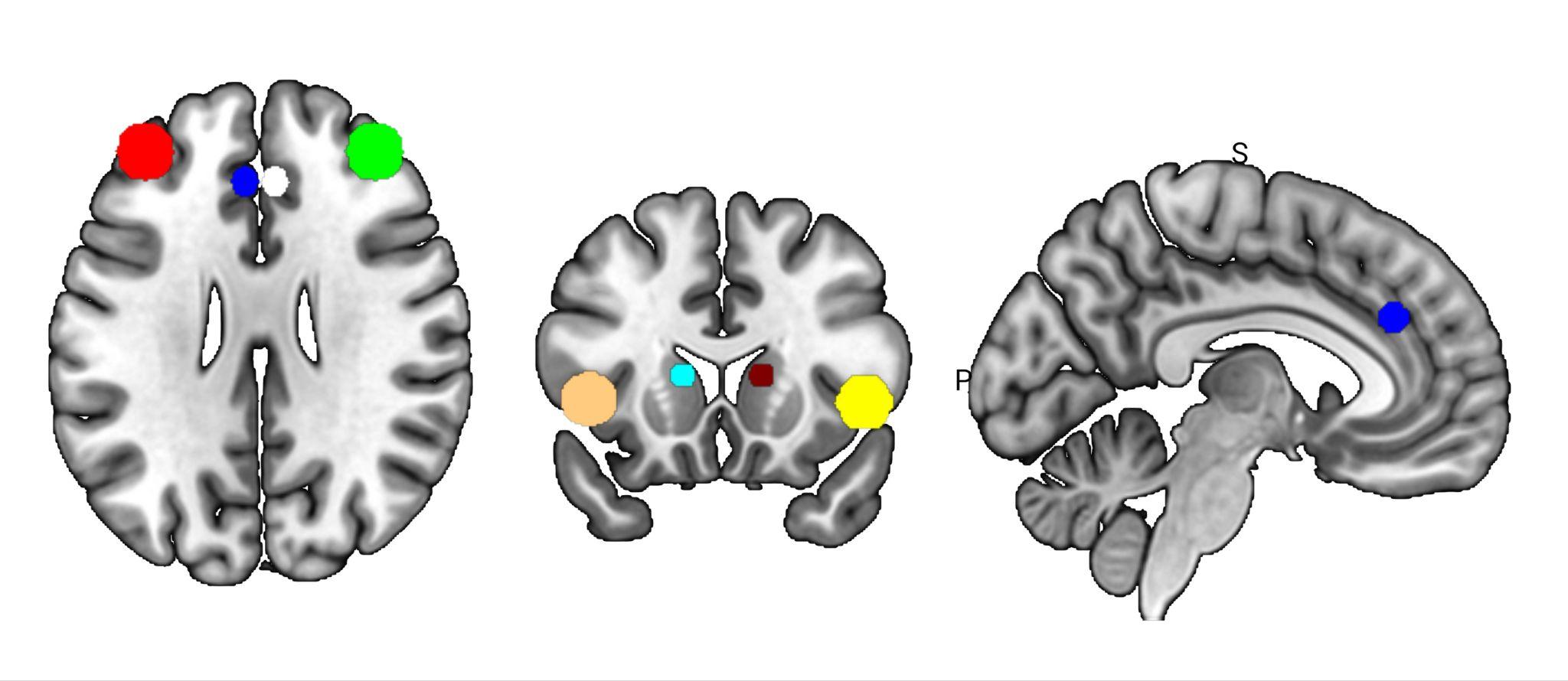


**Figure S1. Location and spherical size of regions-of-interest (ROI).** A spherical ROI with a 10 mm radius was adopted for bilateral DLPFC, as in previous publications from our lab (Chang et al., 2024a, 2024b). Other ROIs had deviating spherical sizes to prevent anatomical overlap. Red = left DLPFC; Green = right DLPFC; Navy = left rostral ACC; White = right rostral ACC; Orange = left anterior insula; Yellow = right anterior insula; Turquoise = left dorsal caudate nucleus; Bordeaux = right dorsal caudate nucleus.

*
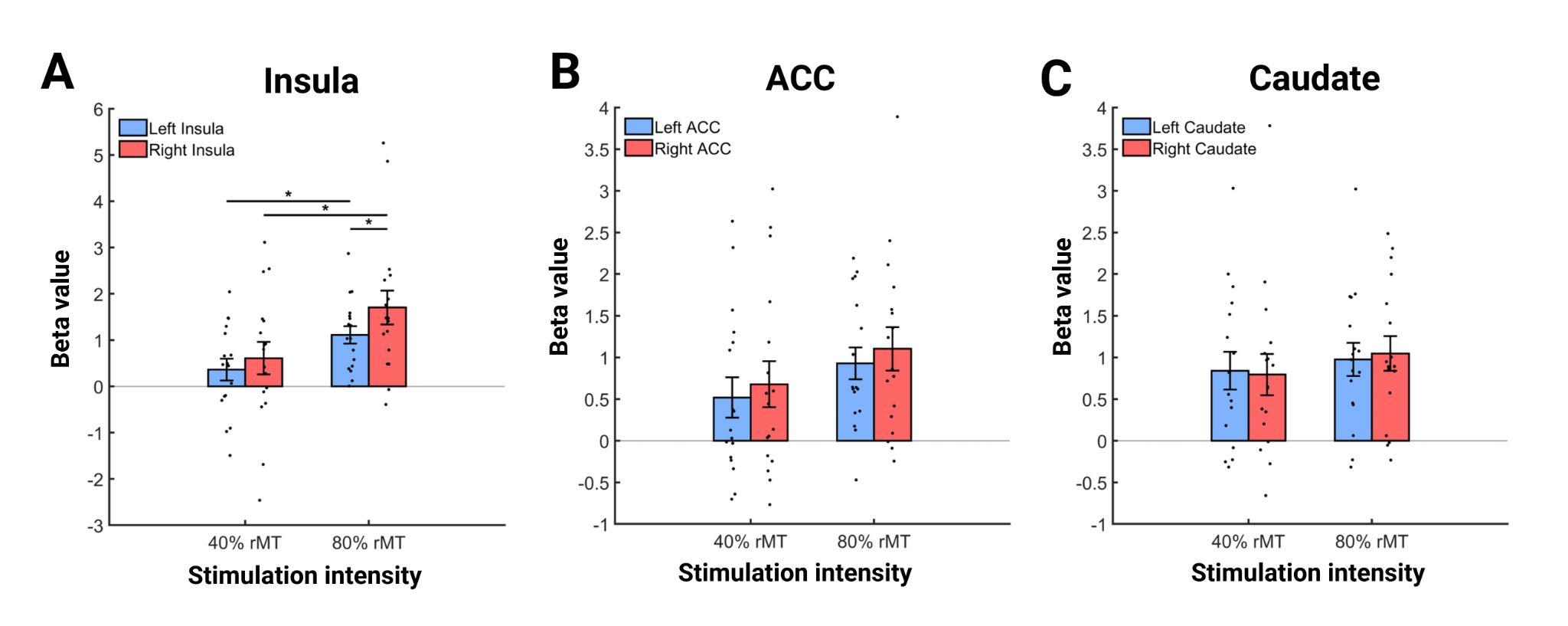
*

**Figure S2. 10 Hz rTMS-evoked BOLD responses in bilateral Insula, ACC, Caudate ROIs at 40% rMT and 80% rMT.** Black dots represent individual subjects. Error bars show ± SEM. * p < .05; ** p < .01; *** p < .001. Corrections for multiple comparisons were applied within each region of interest (ROI), but not across the entire set of ROIs.

*
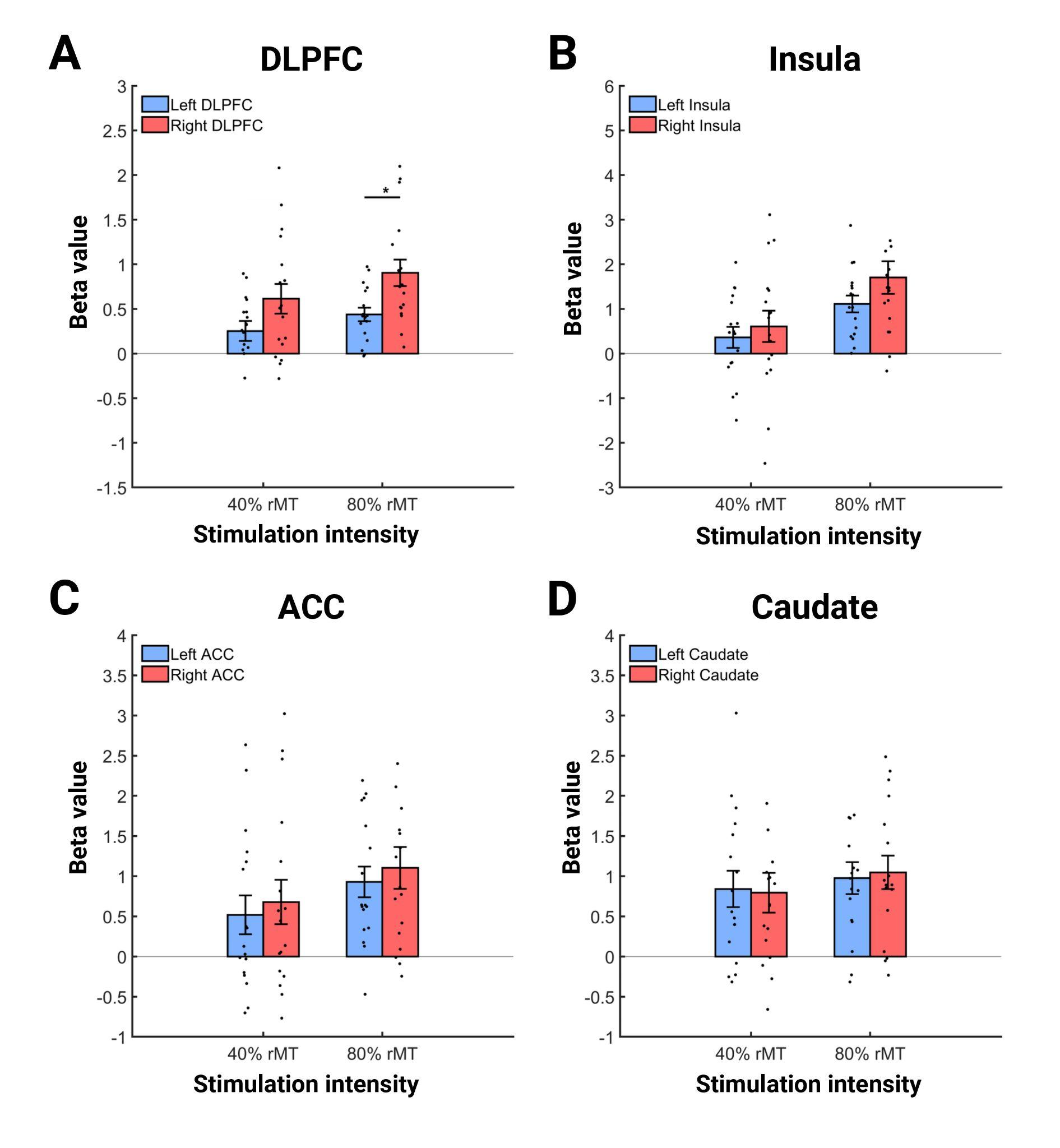
*

**Figure S3. 10 Hz rTMS-evoked BOLD responses in bilateral ROIs at 40% rMT and 80% rMT with outlier removal.** Black dots represent individual subjects. Error bars show ± SEM. * p < .05; ** p < .01; *** p < .001. After removing outliers, statistical significance in the Insula ROIs was removed. The significance in the DLPFC remained robust.


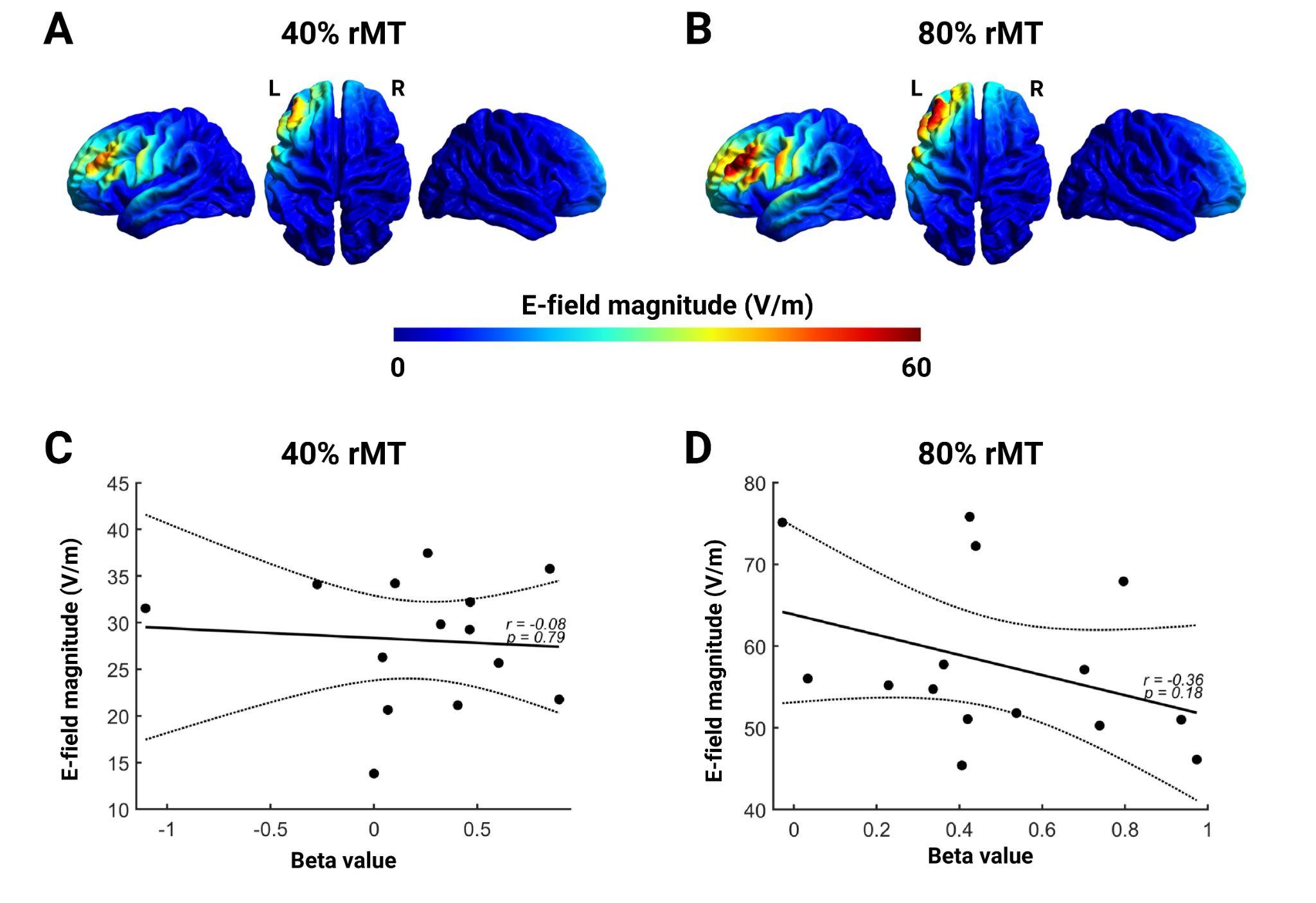
**Figure S4. E-field simulations of 10 Hz rTMS over the left DLPFC.** Mean E-Field strength showed specificity to the targeted location in the left DLPFC at 40% rMT (A) and 80% rMT (B). Individual beta values in the left DLPFC did not correlate with E-field strength at 40% rMT (C) or 80% rMT (D).


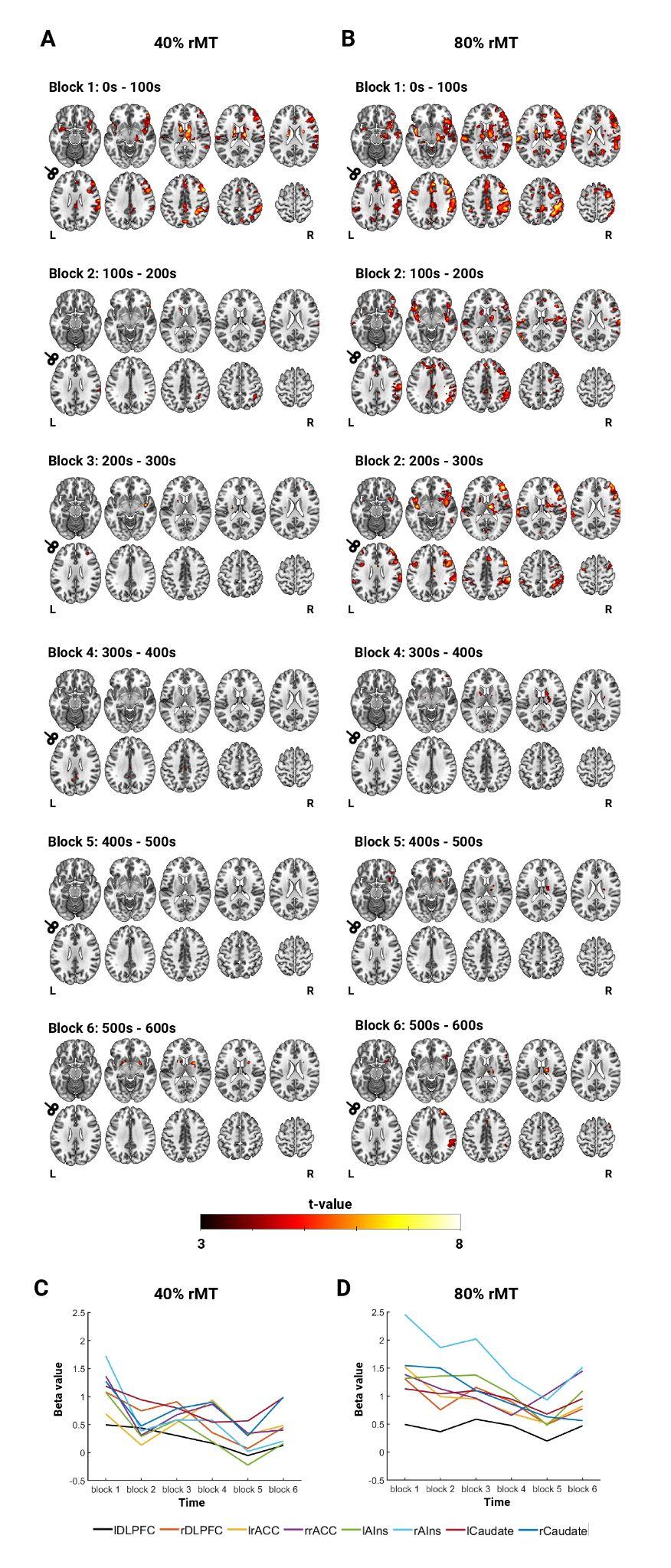


**Figure S5. Temporal dynamics of BOLD response during 10 Hz rTMS protocol.** The 10 Hz rTMS protocol lasted 10 minutes and consisted of 60 trains of 10 Hz rTMS (600 pulses total). To examine how the BOLD response developed over time, the full protocol was divided into six blocks, each containing 10 trains (100 pulses per block). Axial slices Z = -16, -8, 12, 18, 22; 26, 34, 42, 52, 62.


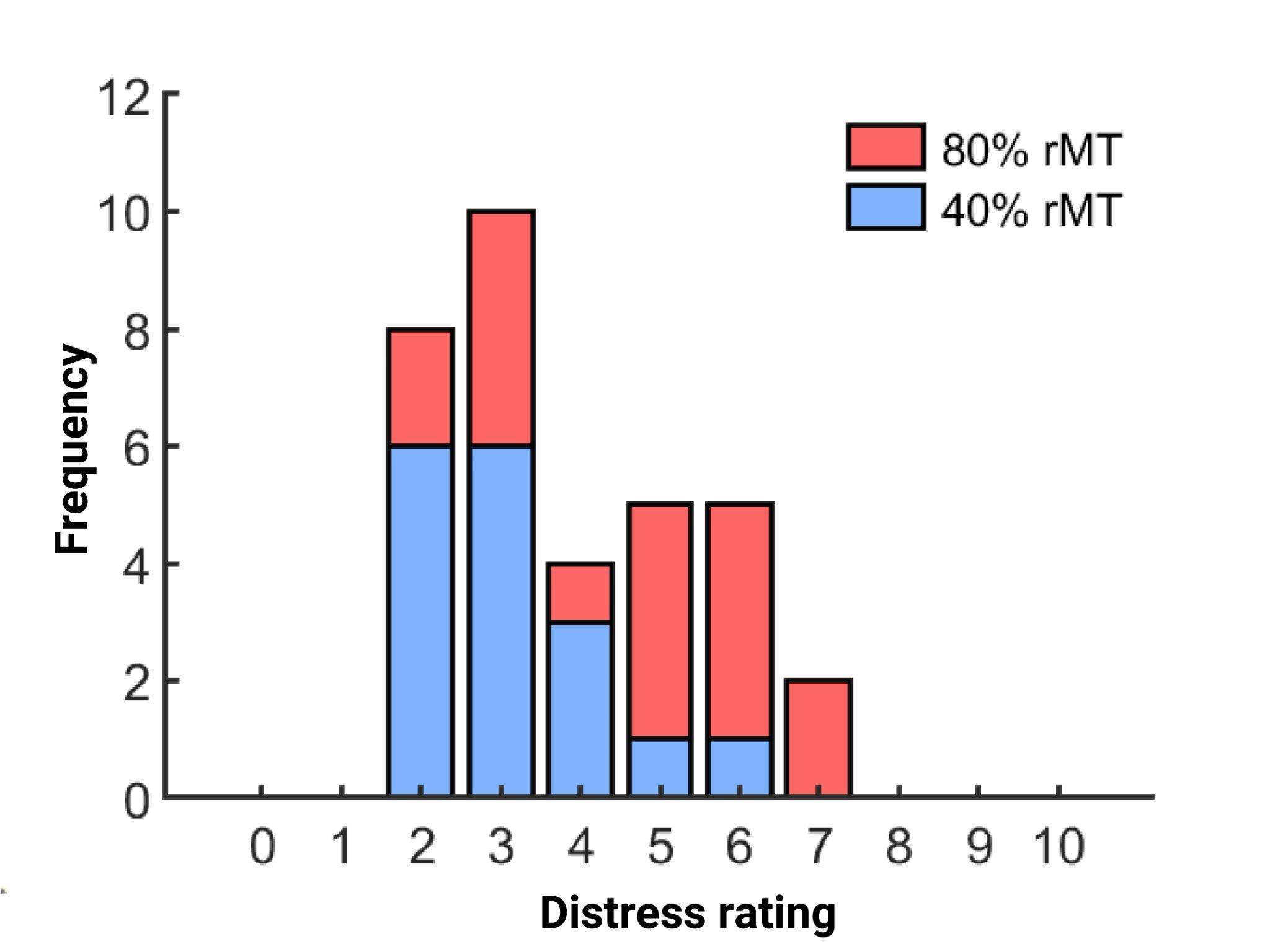
**Figure S6. Distress ratings after TMS-fMRI sessions.** Distress ratings, on a scale of 0 (no stress/pain)10 (extreme stress/pain), were recorded immediately after each stimulation session by asking participants to rate their pain and stress levels during the interleaved TMS-fMRI session. No subjects verbally reported experiencing pain during the experiment. (A) Distribution of distress ratings for both stimulation intensities. Distress ratings were higher for 80% rMT (M = 4.59, SD = 1.61) than for 40% rMT (M = 3.12, SD = 1.13). (B) Distress ratings sorted by session order.

**Appendix. Supplementary References**

Bergmann, T. O., Varatheeswaran, R., Hanlon, C. A., Madsen, K. H., Thielscher, A., & Siebner, H. R. (2021). Concurrent TMS-fMRI for causal network perturbation and proof of target engagement. *NeuroImage*, *237*, 118093.<https://doi.org/10.1016/j.neuroimage.2021.118093>

Blumberger, D. M., Vila-Rodriguez, F., Thorpe, K. E., Feffer, K., Noda, Y., Giacobbe, P., Knyahnytska, Y., Kennedy, S. H., Lam, R. W., Daskalakis, Z. J., & Downar, J. (2018). Effectiveness of theta burst versus high-frequency repetitive transcranial magnetic stimulation in patients with depression (THREE-D): A randomised non-inferiority trial. *Lancet (London, England)*, *391*(10131), 1683–1692.<https://doi.org/10.1016/S0140-6736(18)30295-2>

Chang, K.-Y., Tik, M., Mizutani-Tiebel, Y., Schuler, A.-L., Taylor, P., Campana, M., Vogelmann, U., Huber, B., Dechantsreiter, E., Thielscher, A., Bulubas, L., Padberg, F., & Keeser, D. (2024a). Neural response during prefrontal theta burst stimulation: Interleaved TMS-fMRI of full iTBS protocols. *NeuroImage*, *291*, 120596.<https://doi.org/10.1016/j.neuroimage.2024.120596>

Chang, K.-Y., Tik, M., Mizutani-Tiebel, Y., Taylor, P., Van Hattem, T., Falkai, P., Padberg, F., Bulubas, L., & Keeser, D. (2024b). Dose-Dependent Target Engagement of a Clinical Intermittent Theta Burst Stimulation Protocol: An Interleaved Transcranial Magnetic Stimulation–Functional Magnetic Resonance Imaging Study in Healthy People. *Biological Psychiatry: Cognitive Neuroscience and Neuroimaging*, S2451902224002441.<https://doi.org/10.1016/j.bpsc.2024.08.009>

Griffanti, L., Douaud, G., Bijsterbosch, J., Evangelisti, S., Alfaro-Almagro, F., Glasser, M. F., Duff, E. P., Fitzgibbon, S., Westphal, R., Carone, D., Beckmann, C. F., & Smith, S. M. (2017). Hand classification of fMRI ICA noise components. *NeuroImage*, *154*, 188–205.<https://doi.org/10.1016/j.neuroimage.2016.12.036>

Hanlon, C. A., Canterberry, M., Taylor, J. J., DeVries, W., Li, X., Brown, T. R., & George, M. S. (2013). Probing the frontostriatal loops involved in executive and limbic processing via interleaved TMS and functional MRI at two prefrontal locations: A pilot study. *PloS One*, *8*(7), e67917.

Hawco, C., Voineskos, A. N., Steeves, J. K. E., Dickie, E. W., Viviano, J. D., Downar, J., Blumberger, D. M., & Daskalakis, Z. J. (2018). Spread of activity following TMS is related to intrinsic resting connectivity to the salience network: A concurrent TMS-fMRI study. *Cortex; a Journal Devoted to the Study of the Nervous System and Behavior*, *108*, 160–172. https://doi.org/10.1016/j.cortex.2018.07.010

Jenkinson, M., Beckmann, C. F., Behrens, T. E. J., Woolrich, M. W., & Smith, S. M. (2012). FSL. *NeuroImage*, *62*(2), 782–790.<https://doi.org/10.1016/j.neuroimage.2011.09.015>

Krönke, K.-M., Wolff, M., Shi, Y., Kräplin, A., Smolka, M. N., Bühringer, G., & Goschke, T. (2020). Functional connectivity in a triple-network saliency model is associated with real-life self-control. *Neuropsychologia*, *149*, 107667.<https://doi.org/10.1016/j.neuropsychologia.2020.107667>

Mizutani-Tiebel, Y., Tik, M., Chang, K.-Y., Padberg, F., Soldini, A., Wilkinson, Z., Voon, C. C., Bulubas, L., Windischberger, C., & Keeser, D. (2022). Concurrent TMS-fMRI: Technical Challenges, Developments, and Overview of Previous Studies. *Frontiers in Psychiatry*, *13*, 825205.<https://doi.org/10.3389/fpsyt.2022.825205>

Park, J., Kim, T., Kim, M., Lee, T. Y., & Kwon, J. S. (2020). Functional Connectivity of the Striatum as a Neural Correlate of Symptom Severity in Patient with Obsessive-Compulsive Disorder. *Psychiatry Investigation*, *17*(2), 87–95.<https://doi.org/10.30773/pi.2019.0206>

Peng, Z., He, T., Ren, P., Jin, L., Yang, Q., Xu, C., Wen, R., Chen, J., Wei, Z., Verguts, T., & Chen, Q. (2022). Imbalance between the caudate and putamen connectivity in obsessive-compulsive disorder. *NeuroImage. Clinical*, *35*, 103083.<https://doi.org/10.1016/j.nicl.2022.103083>

Power, J. D., Barnes, K. A., Snyder, A. Z., Schlaggar, B. L., & Petersen, S. E. (2012). Spurious but systematic correlations in functional connectivity MRI networks arise from subject motion. *NeuroImage*, *59*(3), 2142–2154.<https://doi.org/10.1016/j.neuroimage.2011.10.018>

Riddle, J., Scimeca, J. M., Pagnotta, M. F., Inglis, B., Sheltraw, D., Muse-Fisher, C., & D’Esposito, M. (2022). A guide for concurrent TMS-fMRI to investigate functional brain networks. *Frontiers in Human Neuroscience*, *16*, 1050605.<https://doi.org/10.3389/fnhum.2022.1050605>

Thielscher, A., Antunes, A., & Saturnino, G. B. (2015). Field modeling for transcranial magnetic stimulation: A useful tool to understand the physiological effects of TMS? *Annual International Conference of the IEEE Engineering in Medicine and Biology Society. IEEE Engineering in Medicine and Biology Society. Annual International Conference*, *2015*, 222–225.<https://doi.org/10.1109/EMBC.2015.7318340>

Zhou, Y., Shi, L., Cui, X., Wang, S., & Luo, X. (2016). Functional Connectivity of the Caudal Anterior Cingulate Cortex Is Decreased in Autism. *PloS One*, *11*(3), e0151879.<https://doi.org/10.1371/journal.pone.0151879>
